# Surface Engineered Biomolecular Condensates for Targeted Cell Cytotoxicity

**DOI:** 10.1002/advs.202518312

**Published:** 2025-11-25

**Authors:** Chengying Yin, Cheng Wu, Xinran Yu, Yifeng Zhu, Baohu Wu, Yue Wang, Liangfei Tian

**Affiliations:** ^1^ Department of Ambulatory Surgery Women's Hospital School of Medicine Zhejiang University Hangzhou 310027 China; ^2^ Department of Gynecology Women's Hospital School of Medicine Zhejiang University Hangzhou 310027 China; ^3^ Institute of Genetics School of Medicine Zhejiang University Hangzhou 310058 China; ^4^ Zhejiang Key Laboratory of Intelligent Sensing Technology and Advanced Medical Instrument Key Laboratory of Biomedical Engineering of Ministry of Education Department of Biomedical Engineering Zhejiang University Hangzhou 310027 China; ^5^ MLZ JCNS JCNS‐4 Forschungszentrum Jülich Lichtenbergstr. 1 85748 Garching Germany; ^6^ Innovation Center for Smart Medical Technologies & Devices Binjiang Institute of Zhejiang University Hangzhou 310053 China

**Keywords:** biomolecular condensates, cell cytotoxicity, enzymatic reaction, surface engineering

## Abstract

Biomolecular condensates have attracted attention in membraneless cellular organization, bioreactor design, drug delivery, cellular regulation, and tissue engineering. However, the openness of their interfaces poses challenges for precise enzymatic control. Here, a two‐step interfacial engineering strategy is developed to construct a decanoic acid (DA) membrane on decalysine/polyinosinic acid biomolecular condensates. This membrane reduces interfacial mobility. It also enhances enrichment of hydrophobic small molecules (e.g., Nile Red). Crucially, it imposes molecular‐weight‐dependent spatial control over biomacromolecules: species ≤60 kDa (e.g., single‐stranded DNA (ssDNA), Horseradish Peroxidase (HRP), lipase) enrich within the microdroplet interior, while high‐molecular‐weight alkaline phosphatase (ALP) localizes at the interface. This spatial regulation significantly modulates enzymatic kinetics, boosting catalytic activity for both lipase and ALP within DA‐coated condensates. Specifically, interfacial ALP accelerates dephosphorylation of N‐(9‐fluorenylmethoxycarbonyl)‐L‐tyrosine‐(O)‐phosphate (Fmoc‐TyrP) and subsequent nanofiber growth, altering the condensate's internal physical environment and triggering release of enriched biomacromolecules like ssDNA. In cell co‐culture, DA‐coated condensates efficiently deliver ALP on HeLa cell membranes; subsequent Fmoc‐TyrP addition induced apoptosis, reducing cell viability to 5%, compared to 50% with uncoated condensates. This work establishes a foundation for precision biocatalysis and targeted therapeutic platforms using engineered condensates, enabling functional customization of synthetic organelles.

## Graphical abstract

Interfacial engineering of biomolecular condensates with a decanoic acid (DA) membrane enables molecular‐weight‐gated enzyme localization. Small biomolecules (≤60 kDa) enrich internally, while high‐molecular‐weight alkaline phosphatase (ALP) localizes at the interface. This spatio distribution boosts enzymatic activity and triggers in situ nanofiber formation. When applied to cancer cells, DA‐coated condensates deliver ALP to HeLa membranes, inducing targeted apoptosis (5% cell viability vs. 50% in controls).

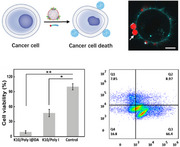

## 1 Introduction

Biomolecular condensates have garnered significant attention in the fields, such as membraneless cellular organization,^[^ ^1^, ^2^, ^3^ ^]^ bioreactor design,^[^ ^4^, ^5^, ^6^, ^7^, ^8^, ^9^ ^]^ drug delivery systems,^[^ ^10^, ^11^, ^12^, ^13^ ^]^ cellular regulation,^[^ ^14^, ^15^, ^16^ ^]^ and tissue engineering^[^ ^17^, ^18^ ^]^ due to their capacity for substance enrichment^[^ ^19^, ^20^, ^21^, ^22^, ^23^, ^24^, ^25^, ^26^ ^]^ and dynamic responsiveness to environmental changes.^[^ ^27^, ^28^, ^29^, ^30^, ^31^, ^32^ ^]^ However, the inherent openness of the interfaces of these condensates presents significant obstacles to the precise regulation of mass exchange processes,^[^ ^14^, ^33^, ^34^ ^]^ ultimately impeding their broader biomedical applications.^[^ ^35^, ^36^, ^37^ ^]^ This challenge becomes particularly intricate when enzymatic reactions are confined within subcellular‐scale biomolecular condensates. Emerging evidence underscores that interfacial properties of biomolecular condensates serve as pivotal determinants governing molecular communication with the external environment.^[^ ^38^, ^39^, ^40^, ^41^, ^42^, ^43^, ^44^, ^45^, ^46^ ^]^ The interfacial layer orchestrates a complex interplay between selective molecular permeation and reaction kinetics,^[^ ^19^, ^47^, ^48^, ^49^, ^50^, ^51^, ^52^, ^53^, ^54^, ^55^, ^56^, ^57^ ^]^ establishing a multifaceted regulatory network that remains largely unexplored. Thus, interfacial engineering of biomolecular condensates to achieve precise enzymatic control stands as a pivotal step in advancing their applications across biomedical research, synthetic biology, and related disciplines.

To tackle this challenge, we introduce a two‐step interface engineering approach to construct a decanoic acid (DA) membrane on the interface of decalysine/polyinosinic acid (K10/Poly I) biomolecular condensates, effectively reducing interface mobility. This DA membrane enhances the enrichment capacity for hydrophobic small molecules (e.g., Nile Red) while demonstrating selectivity for transmembrane substance transport based on molecular weight for biomacromolecules. Lower molecular‐weight biomacromolecules can readily pass through, while large molecular weight biomacromolecules, such as alkaline phosphatase (ALP), are restricted to the biomolecular condensates surface. This regulation of spatial distribution markedly influences enzymatic reaction kinetics. Surface‐localized ALP accelerates in‐situ nanofiber growth, enabling the surfactant‐coated biomolecular condensates to induce HeLa cell apoptosis with a survival rate of 5%, compared to only 50% with the uncoated system. Unlike previous studies on surface‐modified condensates that primarily focused on enhancing stability or permeability control, our work elucidates how a decanoic acid membrane confers molecular‐weight‐dependent sieving behavior and programmable enzyme spatial localization. Our study lays the foundation for the development of a precision biocatalytic system and a targeted therapeutic platform utilizing biomolecular condensates and opens up new possibilities for the functional customization of artificial organelles in synthetic biology.

## 2 Results and Discussion

**Figure 1:**
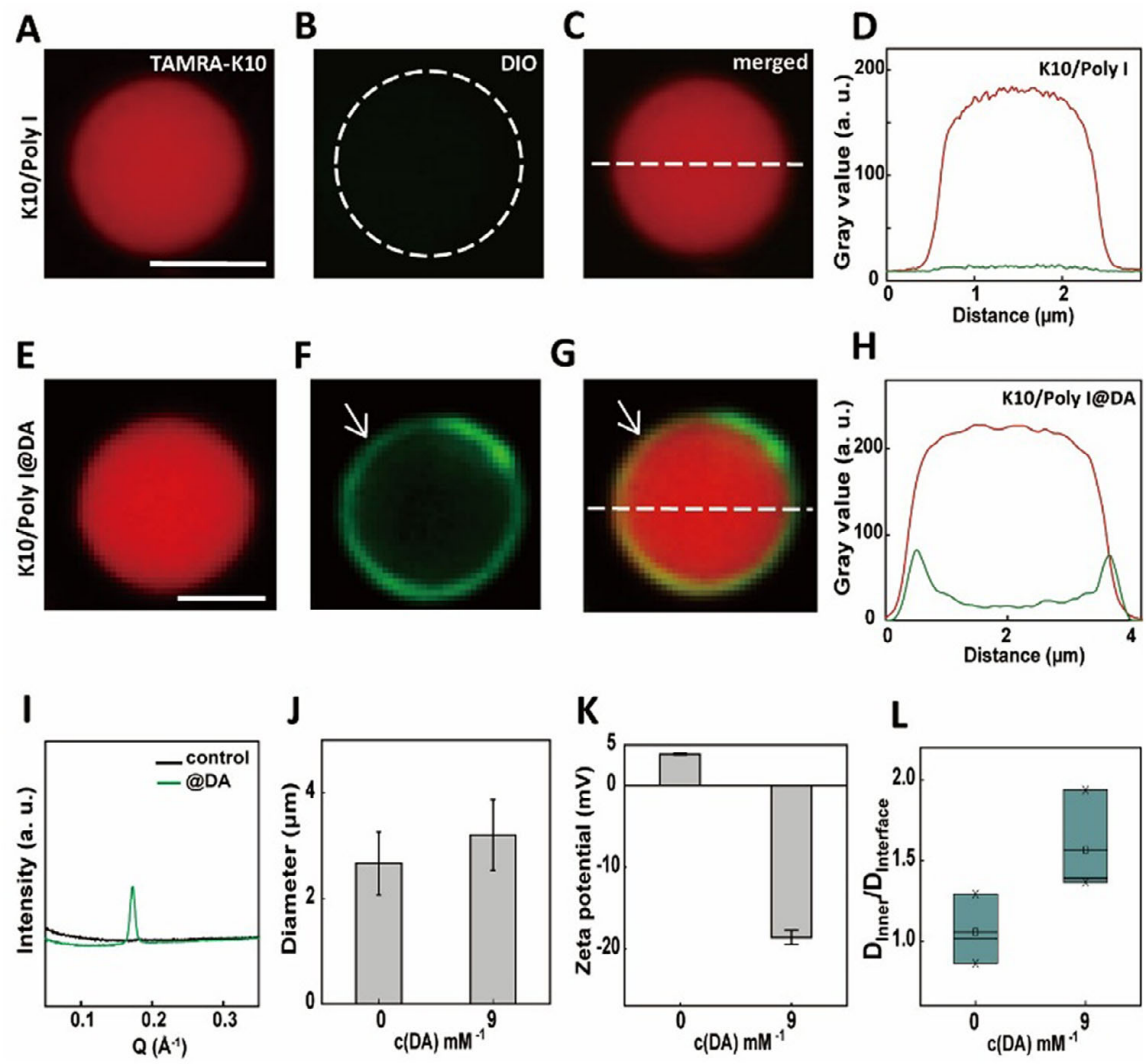
A–H) Confocal fluorescence microscopy images, and corresponding fluorescence intensity (gray values) profiles of DiO (green, 3 µM, λ_ex_ = 488 nm and λ_em_ = 501 nm), TAMRA‐K10 (red, 2 µµ, λ_ex_ = 561 nm and λ_em_ = 580 nm) labeled K10/Poly I condensates before (top row) (A–D) and after (bottom row) (E–H) the addition of DA (final concentration, 9 mm). All scale bars: 2 µm. White arrows in panels F and G highlight the membrane edges formed at the condensate interface. I) SAXS profiles of K10/Poly I condensates suspension as a control (black line), and the condensates suspension after the addition of the DA (final concentration, 9 mm, green line). J) Mean diameter of K10/Poly I condensates before and after the addition of DA (final concentration, 9 mm). The mean diameter of condensates was calculated from confocal microscopy images using ImageJ software. Error bars represent the standard deviation from the average value measured on 20 condensates in two different fields of view of the same sample. K) Zeta potentials of K10/Poly I condensates before and after the addition of DA (final concentration, 9 mm). Error bars indicate the standard deviation of three repeated measurements for zeta potential. L) Plots of the change of diffusion coefficient deduced from FRAP measurements in DA‐coated (final concentration, 9 mm) and non‐coated condensates.

The preparation of DA‐coated K10/Poly I biomolecular condensates has been developed using a two‐step method (Scheme S1, Supporting Information). Specifically, K10 and Poly I were first mixed to prepare a condensate suspension with a final monomer concentration of 5/5 mm (Figure S1, Supporting Information). Subsequent addition of DA (final concentration, 9 mm) to the suspension induced localized interfacial enrichment of the membrane probe benzoxazolium, 3‐octadecyl‐2‐[3‐(3‐octadecyl‐2(3H)‐benzoxazolylidene)‐1‐propenyl]‐perchlorate (DiO), indicating membrane formation at the biomolecular condensates interface (**Figure** **1**A–H; Figure S2, Supporting Information). This observation was further supported by small‐angle X‐ray scattering (SAXS) measurements, which revealed a peak of q = 1.72 nm^−1^, referring to an interlayer distance (d) of 3.7 nm (Figure 1I). Fluorescence‐activated cell sorting (FACS) confirmed no significant change in biomolecular condensate counts following DA addition (Figure S3, Supporting Information). However, membrane formation resulted in no significant difference in droplet diameter (2.6–3.2 µm) when considering measurement error (Figure 1J) and a substantial reversal of ζ‐potential from +3.9 to −18.6 mV (Figure 1K). The formation of the DA membrane is likely driven by a combination of electrostatic and hydrophobic interactions, in agreement with the ζ‐potential and SAXS data. Fluorescence recovery after photobleaching (FRAP) experiments revealed significantly slower fluorescence recovery at the interface compared to internal regions in DA‐coated condensates (Figure 1L; Figure S4, Supporting Information). In contrast, uncoated K10/Poly I condensates exhibited uniform recovery kinetics throughout (Figure 1L; Figure S5, Supporting Information), demonstrating that the DA coating markedly reduces interfacial membrane fluidity.

**Figure 2:**
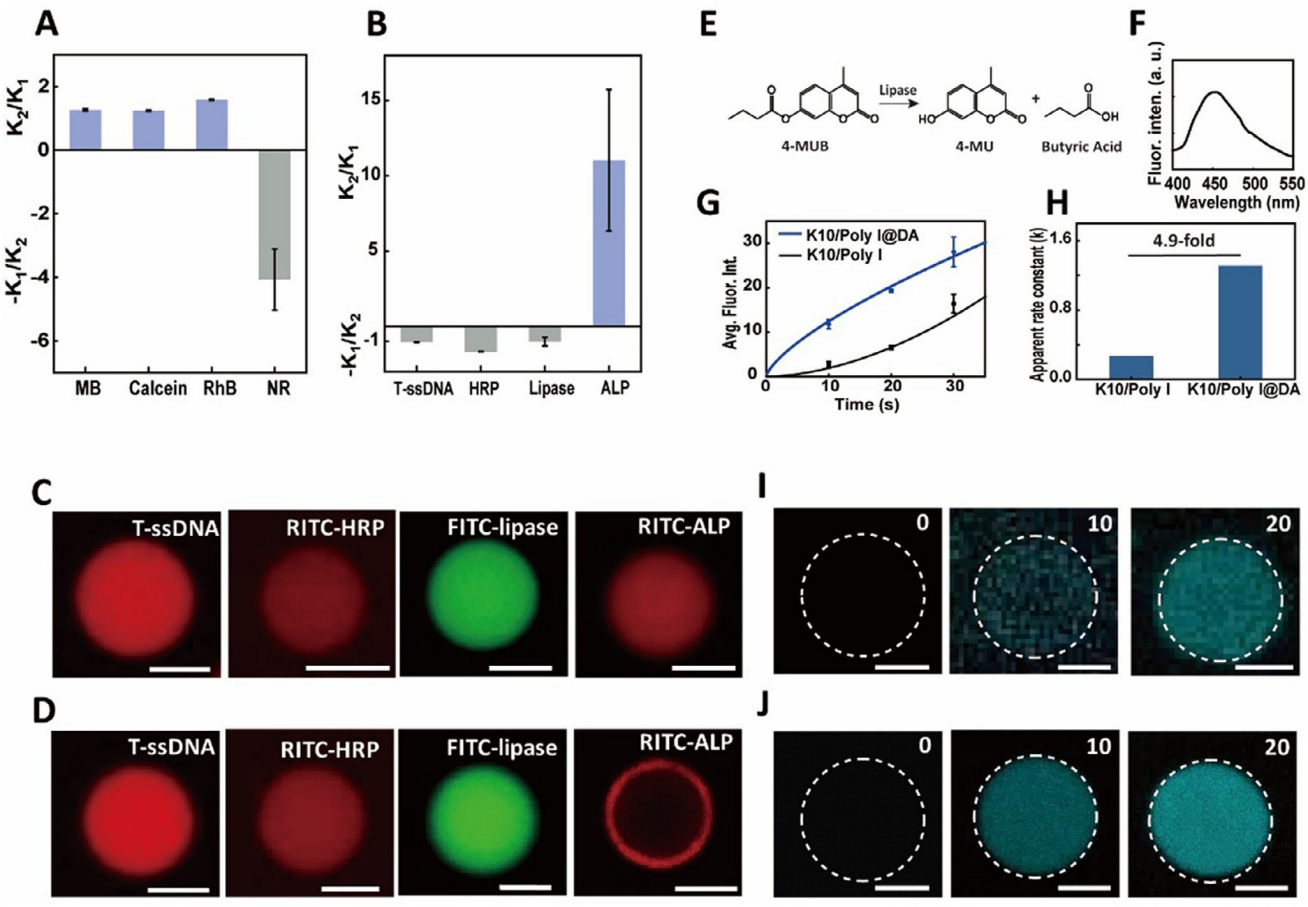
A,B) The plots of the changes of partitioning coefficients for small molecules C) and biomacromolecules D) before and after the addition of DA. K1 represents the partitioning coefficient in DA‐coated condensates, and K2 represents the partitioning coefficient in non‐coated condensates. The difference in the change of partitioning coefficient was obtained by K2/K1 when more dyes were sequestered in non‐coated condensates, or by ‐K1/K2 when more dyes were sequestered in DA‐coated condensates. (C,D) Confocal fluorescence microscopy images of the sequestration of T‐ssDNA (λ_ex_ = 561 nm and λ_em_ = 580 nm), RITC‐HRP (λ_ex_ = 561 nm and λ_em_ = 580 nm), FITC‐lipase (λ_ex_ = 488 nm and λ_em_ = 518 nm), and RITC‐ALP (λ_ex_ = 561 nm and λ_em_ = 580 nm) in K10/Poly I within condensates before (C) and after (D) the addition of DA (final concentration, 9 mm). All scale bars: 2 µm. E,F) The catalytic reaction scheme for the conversion of 4‐MUB (E), and the fluorescence spectrum of the reaction product, 4‐methylumbelliferone (4‐MU) (F). G,H) The time‐dependent average fluorescence intensity plots (G) for 4‐methylumbelliferone (4‐MU, fluorescent, excitation peak: 372 nm, emission peak: 445 nm) and Corresponding plots of the slopes of the initial change of fluorescence intensities (apparent rate constant, k) H) for the conversion of 4‐MUB as shown in (E,F). The apparent rate constants were deduced from the linear fit of the plots of the change of fluorescence intensity for 4‐methylumbelliferone (4‐MU, fluorescent, excitation peak: 372 nm, emission peak: 445 nm) from 0 to 30 s. I, J) Confocal fluorescence microscopy images recorded after addition of 4‐MUB (0.1 mg ml^−1^) to lipase (0.03 U µL^−1^)‐containing K10/Poly I condensates I) and with DA (final concentration, 9 mm) coated K10/Poly I condensates J). All scale bars: 2 µm.

Compared to uncoated K10/Poly I biomolecular condensates, DA‐coated K10/Poly I condensates exhibit significantly altered enrichment behaviors for small molecules and biomacromolecules. While the enrichment of positively, negatively, or zwitterionically charged small molecules (e.g., methylene blue (MB), calcein, and rhodamine B (RhB)) is slightly reduced, the enrichment efficiency for the hydrophobic small molecules Nile Red (NR), N‐(7‐Nitrobenz‐2‐oxa‐1,3‐diazol‐4‐yl)‐1,2‐dihexadecanoyl‐sn‐glycero‐3‐phosphoethanolamine (NBD‐PE), and DiO is enhanced (**Figure** **2**A; Figures S6 and S7, Supporting Information). The differential enrichment of small molecules is likely attributed to the newly formed hydrophobic DA membrane, which enhances the partitioning of hydrophobic molecules like NR, while the altered interfacial properties may slightly reduce the enrichment of charged hydrophilic dyes. Notably, DA coating significantly modifies the spatial distribution profiles of biomacromolecules within the condensates (Figure 2B–D). Specifically, for biomacromolecules with lower molecular weights (e.g., 5‐carboxytetramethylrhodamine‐single‐stranded DNA (T‐ssDNA, 7 kDa), rhodamine isothiocyanate‐horseradish Peroxidase (RITC‐HRP, 40 kDa), and fluorescein isothiocyanate‐lipase (FITC‐lipase, 60 kDa)), DA‐coated biomolecular condensates display a characteristic interior enrichment effect. Compared with uncoated condensates, their enrichment constants show no significant change. In contrast, RITC‐alkaline phosphatase (RITC‐ALP, 140 kDa) predominantly localizes to the interface region of the biomolecular condensates, and its enrichment constant is ≈9% that of the uncoated condensates. In uncoated condensates, all tested biomacromolecules (7–140 kDa) exhibit uniform interior enrichment patterns. This molecular‐weight‐dependent spatial distribution disparity reveals that DA coating confers unique molecular sieving capabilities to biomolecular condensates, enabling selective permeability regulation for biomacromolecules of different sizes. The catalytic activity of lipase within both DA‐coated and uncoated biomolecular condensates was assessed using the fluorescent substrate 4‐methylumbelliferyl butyrate (4‐MUB) (Figure 2E,F). Time‐resolved fluorescence intensity analysis revealed that the initial apparent rate constant (k) of lipase increased by 4.9‐fold in DA‐coated biomolecular condensates, indicating that DA coating enhances the hydrolysis efficiency of lipase toward 4‐MUB (Figures 2G–J).

**Figure 3:**
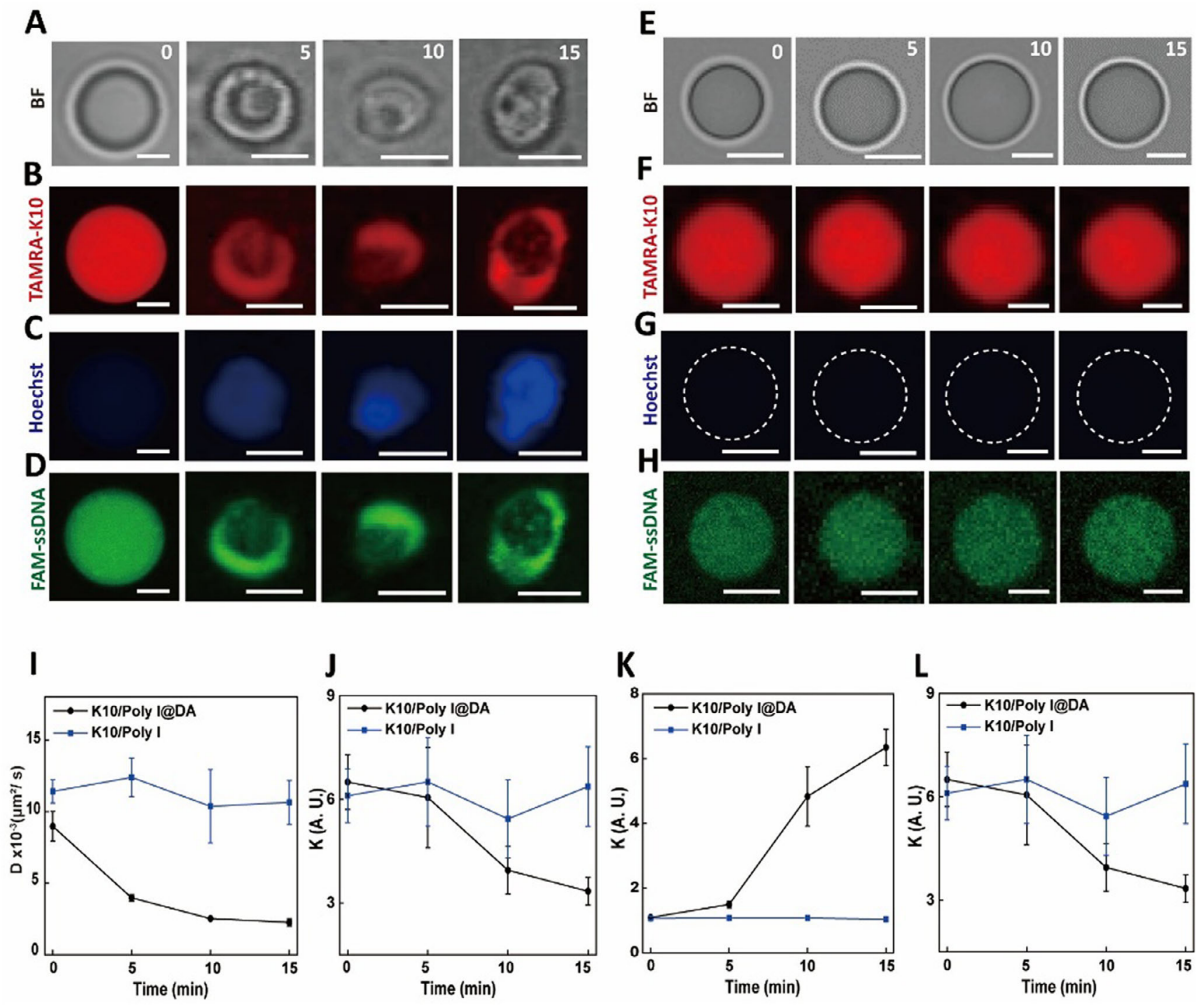
A–H) Time‐dependent optical microscopy images (A) and fluorescence microscopy images (B,C) showing in situ growth of Fmoc‐TyrP nanofilaments within TAMRA‐K10 (red, 2 µµ) labeled DA‐coated K10/Poly I condensates (final concentration, 9 mm) and E–G) TAMRA‐K10 (red, 2 µµ) labeled K10/Poly I condensates. Fluorescence emission is observed upon Hoechst 33 258 (in blue) binding to the growing nanofilaments. (D,H) Its corresponding fluorescence microscopy images showing the spatial distribution of FAM‐ssDNA (green, γ_ex_ = 488 nm, γ_em_ = 500−550 nm) within DA‐coated K10/Poly I condensates (D) and non‐coated K10/Poly I condensates (final concentration, 9 mm) (H). All scale bars, 2 µm. I–L) Its corresponding calculated diffusion coefficients from FRAP Analysis (I), partitioning coefficients (K) of TAMRA‐K10 (J), Hoechst 33 258 (I), and FAM‐ssDNA (L) within DA‐coated K10/Poly I and non‐coated K10/Poly I condensates. Partition coefficients (K) were determined from the ratio of fluorescence intensities in the condensates and in the surrounding dilute aqueous phase. Measurements were performed on 20 condensates, and the average value and standard deviation were calculated.

To further evaluate the catalytic activities of ALP in DA‐coated and uncoated biomolecular condensates, we added phosphorylated amino acid derivative N‐(9‐fluorenylmethoxycarbonyl)‐L‐tyrosine‐(O)‐phosphate (Fmoc‐TyrP) to ALP‐containing DA‐coated and uncoated K10/Poly I condensates (**Figure** **3**; Figure S8, Supporting Information). Under ALP catalysis, Fmoc‐TyrP underwent dephosphorylation and self‐assembled into nanofibrillar structures based on N‐(9‐fluorenylmethoxycarbonyl)‐L‐tyrosine (Fmoc‐TyrOH).^[^ ^58^, ^59^, ^60^, ^61^ ^]^ By performing fluorescent staining of the nanofibers using Hoechst 33258 (which emits blue fluorescence upon binding to nanofibers) and monitoring temporal changes in its fluorescence intensity (Figure 3A–H), the dynamic tracking of Fmoc‐TyrOH nanofiber formation within condensates was achieved. Upon Fmoc‐TyrP addition, ALP‐containing DA‐coated biomolecular condensates exhibited significant morphological deformation, shown by bright field images (Figure 3A) and a decrease in TAMRA‐K10 fluorescent intensity (Figures 3B,J), accompanied by a marked increase in Hoechst 33‐258 fluorescence intensity (Figure 3C,K) and a substantial reduction in microdroplet fluidity (Figure 3I; Figures S9 and S10, Supporting Information). And no significant changes in average mean diameter were observed (Figure S11, Supporting Information). Concurrent with these observations, FAM‐labeled single‐stranded DNA (F‐ssDNA, green fluorescence) underwent redistribution from the interior of the biomolecular condensates and diffusion into the continuous phase (Figures 3D,L). These findings indicate that the formation of an Fmoc‐TyrOH nanofiber within the DA‐coated system could disrupt the condensate phase, elevate the internal viscosity, and ultimately facilitate the release of ss‐DNA into the continuous phase. In contrast, uncoated ALP‐containing K10/Poly I biomolecular condensates showed no significant morphological (Figures 3E–G), or fluorescence intensity changes after Fmoc‐TyrP addition (Figures 3J,K), and microdroplet viscosity remained unchanged (Figure 3J), indicating the absence of nanofibrillation within the condensates.

**Figure 4:**
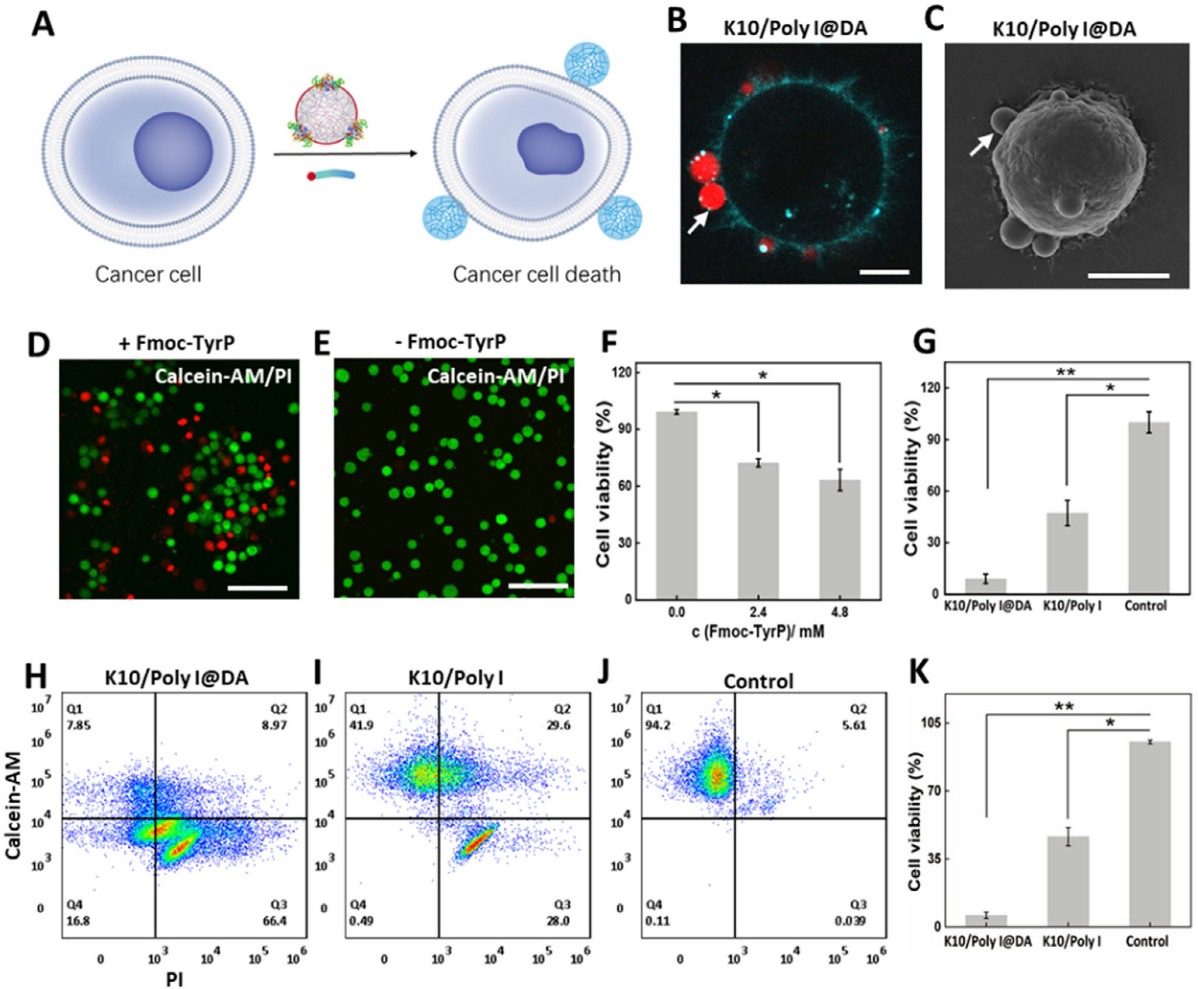
A) Schematic representation of enzyme‐catalyzed formation of nanofibrils to induce cell death. B) Confocal fluorescence microscopy image of CellMask Deep Red Actin Tracking stained HeLa cell treated by DA‐coated K10/Poly I condensates for 15 min. Scale bar: 5 µm. C) SEM image of HeLa cell treated by DA‐coated K10/Poly I condensates for 15 min. Scale bar: 5 µm. D,E) Confocal fluorescence microscopy images of Calcein‐AM and PI‐stained HeLa cells treated by DA‐coated K10/Poly I condensates after the addition of Fmoc‐TyrP (final concentration, 2.4 mm) or without the addition of Fmoc‐TyrP as a control for 15 min. Scale bars: 100 µm. F) Corresponding cell viability plots after the addition of Fmoc‐TyrP (final concentration, 0, 2.4, and 4.8 mm) to HeLa cells treated by ALP containing DA‐coated K10/Poly I condensates. G) Cytotoxicity plots of DA‐coated and non‐coated K10/Poly I condensates by CCK8 assays after incubation with HeLa cells with the addition of Fmoc‐TyrP (final concentration, 2.4 mm) for 24 h. (H‐K) The flow cytometry results of the HeLa cells staining with Calcein‐AM/PI after incubation with DA‐coated and non‐coated K10/Poly I biomolecular condensates with the addition of Fmoc‐TyrP (final concentration, 2.4 mm) for 15 min, and the corresponding data analysis K). The enhanced apoptosis promoted by the coated‐sample confirms the significant effect in cancer‐cell killing. For cell viability assays, *p* < 0.05 was considered statistically significant and *p* < 0.01 was considered highly significant. Error bars indicate the standard deviation of three repeated measurements.

Based on these experimental findings, co‐culture experiments were further conducted involving HeLa cells with ALP‐containing DA‐coated and uncoated condensates (**Figure** **4**A). In contrast to the untreated HeLa cells (Figure S12, Supporting Information), both DA‐coated and uncoated condensates adhered to the cell membrane within 15 min (Figures 4B,C; Figures S13–S18, Supporting Information). The overall surface morphology of the treated HeLa cells showed no apparent differences compared to the untreated cells. Upon the addition of Fmoc‐TyrP to the co‐culture system, significant cell death occurred in the experimental group comprising DA‐coated ALP‐containing condensates. Moreover, with an increase in the concentration of Fmoc‐TyrP, a marked decline in cell viability was noted (Figures 4D–F). In contrast, the experimental group utilizing uncoated K10/Poly I biomolecular condensates containing ALP exhibited less pronounced cell death. To further quantify the disparity in cytotoxicity, the CCK‐8 assay was employed. Following the introduction of Fmoc‐TyrP (2.4 mm), the cell viability in the DA‐coated biomolecular condensates group was significantly reduced compared to the uncoated ALP‐containing biomolecular condensates, dropping to ≈5% after 24 h of co‐culture. Conversely, the cell viability in the uncoated group remained at ≈50%. The cell viability in the groups treated solely with the substrate and the untreated control groups was ≈100% (Figure 4G). Moreover, the results from flow cytometry were consistent with those observed via confocal microscopy, both validating the significant cytotoxic effect of ALP‐containing DA‐coated condensates on cells following the addition of Fmoc‐TyrP (Figures 4H–K). Through Annexin V‐FITC/PI staining and flow cytometry analysis of HeLa cells, it was found that the coated samples significantly promoted cell apoptosis, thereby further highlighting their potential application in killing cancer cells (Figure S19, Supporting Information).

## 3 Conclusion

In summary, this study establishes a two‐step method for constructing a DA membrane on the surface of K10/Poly I biomolecular condensates, significantly reducing interfacial fluidity. This DA membrane enhances the enrichment capacity for hydrophobic small molecules (e.g., Nile Red) while inducing a molecular weight‐dependent spatial distribution for biomacromolecules: lower molecular‐weight species (≤60 kDa; e.g., T‐ssDNA, RITC‐HRP, FITC‐lipase) primarily enrich within the interior of condensates, whereas the high molecular weight ALP localizes predominantly at the interface of the biomolecular condensates. Compared to uncoated systems, the catalytic activity of lipase increased by 4.9‐fold within DA‐coated biomolecular condensates with a similar enrichment constant. Despite exhibiting only 9% of the ALP enrichment observed in uncoated biomolecular condensates, DA‐coated condensates exclusively support ALP‐catalyzed dephosphorylation of Fmoc‐TyrP and subsequent nanofiber formation. This process alters the condensates’ physical microenvironment and triggers the release of enriched biomacromolecules, including single‐stranded DNA. In cell co‐culture experiments, DA‐coated condensates efficiently deliver enzymes like ALP to the proximity of the HeLa cell membrane. Subsequent addition of Fmoc‐TyrP induced apoptosis and reduced cell viability to ≈5%. These findings not only elucidate the cascade regulatory mechanisms linking interfacial architecture, molecular transport, and functional output within biomolecular condensates, but also provide insights into the structure‐function relationship that spans from molecular‐scale interfacial design to macroscopic biological effects. Furthermore, this interfacial engineering strategy may be applicable to neutral or anionic condensates by tailoring amphiphile design, offering a general approach to customize condensate interfaces. Collectively, our findings demonstrate that fatty acid interfacial engineering can endow condensates with membrane‐like molecular sieving, enzyme spatial programmability, and targeted cell‐interaction capabilities, thereby expanding the functional landscape of synthetic organelles beyond conventional permeability control.This framework provides a conceptual pathway for developing precision biocatalytic systems and targeted cancer therapeutics based on biomolecular condensates.

## Conflict of Interest

The authors declare no conflict of interest.

## Author Contributions

C.Y. and C.W. contributed equally to this work. L.T. conceptualized and supervised the project. C.Y. and W.C. contributed to the preparation of cells. C.Y., W.C., X.Y., B.W., and Y.Z. undertook all other experiments; all the authors undertook the data analysis. C.Y., Y.W., and L.T. contributed to the preparation of the manuscript. C.Y. and L.T. wrote the manuscript.

## Supporting information



Supporting Information

## Data Availability

The data that support the findings of this study are available from the corresponding author upon reasonable request.
