# Supplementary material for: Surface Engineered Biomolecular Condensates for Targeted Cell Cytotoxicity
Source: Adv Sci (Weinh). 2025 Nov 25;13(8):e18312. doi: 10.1002/advs.202518312 (PMC12884760; doi:10.1002/advs.202518312)
Supplement: Supplementary file 1 — Supporting Information [file ADVS-13-e18312-s001.docx]

**Supporting information for**

Surface Engineered Biomolecular Condensates for Targeted Cell Cytotoxicity

Chengying Yin^1^^#^, Cheng Wu^2,3#^, Xinran Yu^4^, Yifeng Zhu^4^, Baohu Wu^5^, Yue Wang^1^, Liangfei Tian^1,4,6*^

1. Department of Ambulatory Surgery, Women's Hospital, School of Medicine, Zhejiang University, Hangzhou, 310027, China

2. Department of Gynecology, Women's Hospital, School of Medicine, Zhejiang University, Hangzhou, 310027, China

3. Institute of Genetics, School of Medicine, Zhejiang University, Hangzhou, 310058, China

4. Zhejiang Key Laboratory of Intelligent Sensing Technology and Advanced Medical Instrument, Key Laboratory of Biomedical Engineering of Ministry of Education, Department of Biomedical Engineering, Zhejiang University, Hangzhou, 310027, China

5. MLZ, JCNS, JCNS-4, Forschungszentrum Jülich, Lichtenbergstr. 1, 85748 Garching, Germany

6. Innovation Center for Smart Medical Technologies & Devices, Binjiang Institute of Zhejiang University, Hangzhou, 310053, China

^#^C.Y. and C.W. contributed equally to this work

Correspondence to: [liangfei.tian@zju.edu.cn](mailto:liangfei.tian@zju.edu.cn)

1. **Materials and methods**

**Materials.** Decalysine hydrochloride (K_10_), *N*-terminal TAMRA-labelled K10 (TAMRA-K10), and *N*-terminal FAM-labelled K10 (FAM-K10) were purchased from BankPeptide and used without further purification. Polyinosinic acid potassium salt (Poly I, homopolymer) was purchased from Sigma-Aldrich. Fmoc-TyrP was purchased from njpeptide. Alkaline phosphatase (ALP, ≥10 DEA units/mg) was purchased from Macklin. 4-Methylumbelliferyl butyrate and lipase (from Aspergillus oryzae, 300,000 U/g) were purchased from Aladdin. Calcein, Nile red, and Rhodamine B were purchased from Shanghai Yuanye Bio-Technology Co., Ltd. FAM-ssDNA (5′-ACCACTGAGATCCGGCTGCTAAC-3’, Mw=7531.21 g/mol) and 5-carboxytetramethylrhodamine-single-stranded DNA (TAMRA-ssDNA) (5’-GTTAGCAGCCGGATCTCAGTGGT-3’, Mw=7717.26 g/mol) was obtained from Sangon Biotech. 3,3′-Dioctadecyloxacarbocyanine perchlorate (DiO) was purchased from Beyotime. Decanoic acid was purchased from TCI Shanghai. Milli-Q water (18.2 MΩ·cm) was used throughout the experiments.

**Preparation of K10/Poly I biomolecular condensates.** K10/Poly I biomolecular condensates were produced by mixing K10 (monomer concentration 100 mM, 50 μL, pH 8) and Poly I (monomer concentration 100 mM, 50 μL, pH 8) aqueous solutions in water (900 μL, pH 8) to reach final monomer concentrations of 5 mM for K10 and 5 mM for Poly I. To prepare decanoic acid-coated (DA-coated) biomolecular condensates, a given volume of DA (100 mM, 10 μL) to K10/Poly I biomolecular condensates suspension (100 μL) to reach a final concentration of 9 mM decanoic acid.

**Enzyme modification.** Rhodamine B isothiocyanate (RITC)-labelled alkaline phosphatase (ALP) and fluorescein isothiocyanate (FITC)-labelled lipase was prepared as follows. The enzyme (ALP, 10 mL, 4 mg/mL) or (lipase, 10 mL, 4 mg/mL) were dissolved in sodium carbonate buffered solutions (100 mM, pH 8), and then a dimethyl sulfoxide (DMSO) solution of RITC (200 μL, 2 mg/mL) or FITC (200 μL, 2 mg/mL) was added. The reaction mixtures were kept at 4 °C for 12 h, and then dialysed (molecular weight cut 15 kDa) against Milli-Q water over three days with regular water changes. The fluorescently tagged enzymes were then lyophilized and stored in the dark before use.

**Lipase based enzymatic reaction in condensates.** For the enzyme reaction experiments in K10/Poly I biomolecular condensates, FITC-lipase (0.1%) doped lipase (0.03 U/μL) was pre-sequestered in freshly prepared biomolecular condensates suspension (100 μL). Then, 4-methylumbelliferyl Butyrate (4-MUB, 1 μL, 10 mg/ml, in DMSO) was added to the lipase-containing biomolecular condensates suspension, and immediately loaded in a custom-made capillary slide and imaged by confocal fluorescence microscope. For the enzyme reaction experiment in DA-coated biomolecular condensates, DA (10 μL, 100 mM) were added to the lipase-containing biomolecular condensates suspension which were prepared as described above. The mixtures were incubated for 10 minutes. Then 4-MUB (1 μL, 10 mg/ml, in DMSO) was added to the lipase-containing DA-loaded suspension, and immediately loaded in a custom-made capillary slide and imaged by confocal fluorescence microscope.

**ALP based enzymatic reaction in condensates.** For the enzyme reaction experiments in DA coated- K10/Poly I biomolecular condensates, RITC-ALP (0.1%) doped ALP (0.01 U/μL) was added to freshly prepared biomolecular condensates suspension (100 μL). Then, *N*-fluorenyl-methoxycarbonyl-tyrosine-(*O*)-phosphate (Fmoc-TryP) (10 μL, 100 mM) at pH 9.2 was added to the ALP-containing biomolecular condensates suspension, and immediately loaded in a custom-made capillary slide and imaged by confocal optical and fluorescence microscopy. For the enzyme reaction in non-coated biomolecular condensates as control experiment, Fmoc-TryP (10 μL, 100 mM) at pH 9.2 was added to the non-coated ALP-containing biomolecular condensates suspension, and immediately loaded in a custom-made capillary slide and imaged by confocal fluorescence microscope.

**Calcein-AM/PI cell viability assay.** Calcein-AM/PI staining, simultaneous fluorescence staining of viable and dead cells, was used to assess the cell viability. Briefly, the HeLa cells were seeded in 35 mm confocal laser culture dishes at a density of 3x10^6^ cells mL^−1^, cells were firstly treated with ALP containing- biomolecular condensates, then Fmoc-TyrP at different concentrations (0, 2.4 and 4.8 mM) was added to the mixtures, and incubated for 15 min. Subsequently, calcein-AM solution (final concentration, 5 μM) and PI solution (final concentration, 5 μM) were added into the cell suspension and incubated for 30 min before imaging. The stained cells were imaged on a confocal laser scanning microscope with 20 × objectives. Calcein-AM fluorescence was collected in the range of 505-525 nm with excitation at 488 nm and PI fluorescence was collected above 610 nm with excitation at 543 nm.

***In vitro* cell cytotoxicity.** For cell viability analysis, the HeLa cells (3 × 10^5^ cells/mL) were first treated with ALP containing- non-coated or DA coated- K10/Poly I biomolecular condensates, then add Fmoc-TyrP (final concentration, 2.4 mM), and incubated for 15 min. And then, washing cells three times, staining cells (100 µL) using calcein-AM solution (final concentration, 5 μM) and PI solution (final concentration, 5 μM) in dark at room temperature for 30 min. After staining, the cells were analysed under a flow cytometry (Acen NovoCyte™).

**CCK8 assays.** For cell viability analysis, the HeLa cells were seeded into 96-well plates with a density of 3000 cells per well. And then, the cells were treated with ALP containing non-coated or DA coated- K10/Poly I biomolecular condensates, then Fmoc-TyrP (final concentration, 2.4 mM) was added to the mixtures. After incubation for 0, 24 h, a mixed solution consisting of CCK-8 (10 µL, Solarbio) was added to each well and incubated for an additional 2 h at 37 °C and 5% CO_2_. Finally, the absorbance at 450 nm was measured by a microplate reader (DeTie, HBS-SCANY). The assay was performed in sets of 3 for precision and reproducibility.

**Cell apoptosis analysis.** For cell apoptosis analysis, the HeLa cells were treated with ALP containing- non-coated or DA coated- K10/Poly I biomolecular condensates, then Fmoc-TyrP (final concentration, 2.4 mM) was added to the mixtures.), and incubated for 15 min. And then, the cells were incubated with 5 µL annexin V (final concentration, 1 mg/ml) and 5 µL PI (final concentration, 1 mg/ml) in dark at room temperature for 15 min. After staining, the percentage of apoptotic cells was analysed by flow cytometry (Acen NovoCyte™). The Q2 region represents late apoptotic cells, and Q4 region represents early apoptotic cells.

**K10/Poly I biomolecular condensates/cell cytoskeletal co-localization.** Following 15 min fixation with 4% paraformaldehyde (PFA) at room temperature, HeLa cells were washed three times with phosphate-buffered saline (PBS) and subjected to centrifugation. Subsequent processing involved resuspension in complete culture medium (100 μL) followed by co-incubation with the addition of 20 μL of FAM-labeled K10/Poly I biomolecular condensates. Then with the addition of 80 μL complete medium to achieve a total reaction volume of 200 μL. Then RITC-phalloidin (final concentration, 3 μM) was added for cytoskeletal staining. The suspensions were transferred to confocal imaging dishes and incubated at 37°C for 30 min in a humidified atmosphere containing 5% CO₂. Then samples were analyzed using a confocal microscope equipped with 20× and 100× objectives. Fluorescence signals were acquired under the following spectral conditions: FAM-K10: excitation 488 nm (argon laser), emission collected through 505-525 nm; RITC-phalloidin: Excitation 561 nm (HeNe laser), emission detected using long-pass filter >610 nm.

**Confocal microscopy.** Confocal microscopy images were taken using an OLYMPUS FV3000 laser scanning confocal inverted microscope (LSCM) with FV31S-SW Viewer software and an HCX PL APO CS 100.0×/1.40 oil UV objective, and dye molecules were excited by using specific lasers with the following excitation (λ_ex_) and emission wavelengths (λ_em_): calcein, λ_ex_ = 488 nm and λ_em_ = 518 nm. RITC-, TAMRA-, nile red, rhodamine B, λ_ex_ = 561 nm and λ_em_ = 580 nm. Methylene blue, λ_ex_ = 640 nm and λ_em_ = 680 nm. Fluorescence intensity data was acquired from raw fluorescence images using ImageJ. Partition coefficients (K) were determined from the ratio of fluorescence intensities in the droplets and in the continuous aqueous phase. K1 represent partitioning coefficient in DA-coated condensates, and K2 represent partitioning coefficient in non-coated condensates. The difference in the change of partitioning coefficient was obtained by K2/K1 when more dyes were sequestered in non-coated condensates, or by -K1/K2 when more dyes were sequestered in DA-coated condensates.

**Fluorescence recovery after photobleaching measurements.** The fluorescence recovery after photobleaching (FRAP) measurements were performed on an Olympus FV3000 confocal laser scanning microscope. For coated- K10/Poly I condensates, DiO (interface) was excited with a laser at 488 nm. The emission signals were collected in the range of 490-535 nm. The images were taken before photobleaching using a 488 nm laser at 0.8% intensity for 2 frames (3 s/frame). Bleaching of the condensates was achieved using a 488 nm laser at 30% intensity for 200 ms. After photobleaching, the florescence images were recorded at 0.8% intensity for 35 frames (3 s/frame). Similarly, Methylene blue (inner part) was excited with a laser at 640 nm. The images were taken before photobleaching at 0.8% intensity for 2 frames (3 s/frame). Bleaching of the condensates was achieved using a 640 nm laser at 30% intensity for 200 ms. After photobleaching, the florescence images were recorded at 0.8% intensity for 35 frames (3 s/frame). For non-coated K10/Poly I condensates, Methylene blue (inner part and interface) was excited with a laser at 640 nm. The images were taken before photobleaching at 0.8% intensity for 2 frames (3 s/frame). Bleaching of the condensates was achieved using a 640 nm laser at 30% intensity for 200 ms. After photobleaching, the florescence images were recorded at 0.8% intensity for 35 frames (3 s/frame).

**Small angle X-ray scattering measurements.** SAXS experiments were performed at the KWS-X laboratory beamline (XENOCS XEUSS XL) at JCNS-MLZ, Germany. The coacervate microdroplet suspensions prepared as described previously without further treatment were loaded into thin glass capillaries (inner diameter: 1.5 mm) and analyzed using a high-flux metal-jet source (Ga source, 70 keV).

**Scanning electron microscopy.** Fresh specimens were fixed in 2.5% glutaraldehyde at room temperature for 1-2 h, followed by 0.5 h fixation at 4°C. After removing fixative, samples were rinsed thrice (10-15 min each) with freshly prepared 0.1 M phosphate-buffered saline (PBS, pH 7.4). Sequential dehydration was performed using graded ethanol solutions (30%, 50%, 70%, 90%; 15 min per concentration), followed by two 20-min treatments with absolute ethanol. Critical point drying was conducted prior to gold-palladium sputter-coating using a Leica ACE200 system. Scanning electron microscopy (SEM) imaging was performed with a Nova Nano 450 SEM at 3 kV acceleration voltage.

**Dynamic light scattering (DLS) measurements.** Dynamic light scattering (DLS) measurements were performed using a Zetasizer Nano-ZS instrument (Malvern) equipped with a 633 nm laser. Three measurements were performed on the same sample for each condition, and a representative size distribution reported. Samples were prepared as described previously without further treatment.

**Fluorescence Spectroscopy.** Fluorescence spectra of the reaction product, 4-methylumbelliferone (4-MU) were recorded using a RF-6000 (SHIMADZU CORPORATION) fluorometer with an excitation wavelength of 372 nm. The emission was scanned from 400 to 550 nm in 1 nm steps. The reaction mixture containing 4-MUB (0.1 mg/mL, in DMSO) and lipase (0.03 U/μL) in water (pH 8) was incubated at 25 °C for 15 min.

**Statistical Analysis.** All quantitative experiments were performed in at least three independent replicates. Data are presented as the mean ± standard deviation (SD). For data derived from microscopy images (e.g., condensate average diameter, fluorescence intensity, partitioning coefficients), measurements were taken from a minimum of 15 individual condensates across at least two different fields of view per sample, and the mean values with SD were calculated using ImageJ software. Zeta potential measurements and the diffusion coefficients from Fluorescence Recovery After Photobleaching (FRAP) experiments were repeated three times for each sample, with results reported as mean ± SD. Enzymatic kinetics data were obtained by monitoring fluorescence intensity over time, and the apparent rate constants (k) were deduced from the linear regression of the initial linear phase (0–30 s). For cell viability assays Calcein AM/PI Live/Dead Double Staining, CCK-8 and flow cytometry analysis, statistical significance between two groups was evaluated using a t-test (two-sided testing) in Origin software, with p < 0.05 considered significant and p < 0.01 considered highly significant.

1. **Supplementary figures**


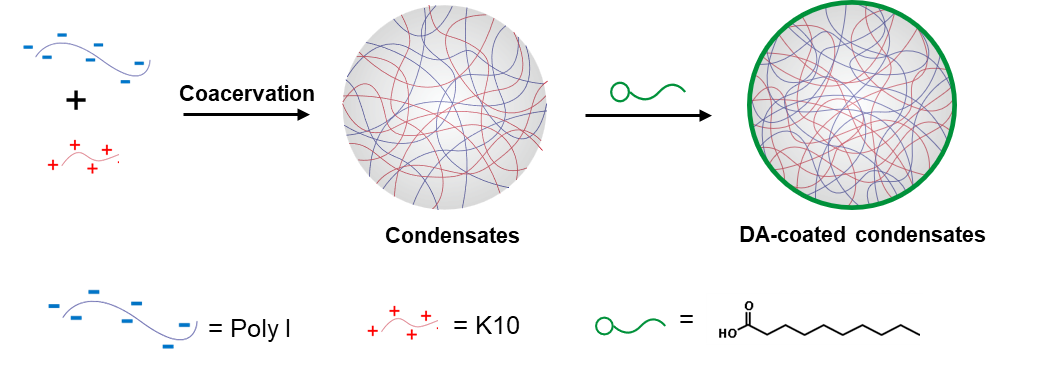


**Scheme S1**. A two-step procedure was developed to prepare DA-coated condensates. In the first step, condensates were formed by direct mixing of aqueous solutions of K10 and Poly I. DA was then added to the resulting condensate suspension, leading to the formation of DA-coated condensates.


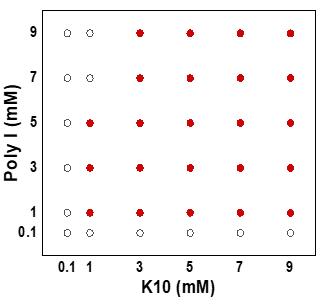


**Figure S1**. The phase diagram of the mixture of K10 and Poly I. The subsequent panels highlight interaction of K10 with different concentrations of Poly I leading to uniform solution (empty circles) or biomolecular condensates (red solid circles). Phase diagram a was obtained over analysis of at least three independent trials.

*
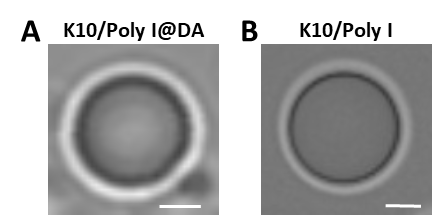
*

**Figure S2.** (**A**, **B**) Optical microscopy images of DA-coated K10/Poly I condensates (final concentration, 9 mM) (**A**) and (**B**) K10/Poly I condensates. Scale bars, 1 μm.


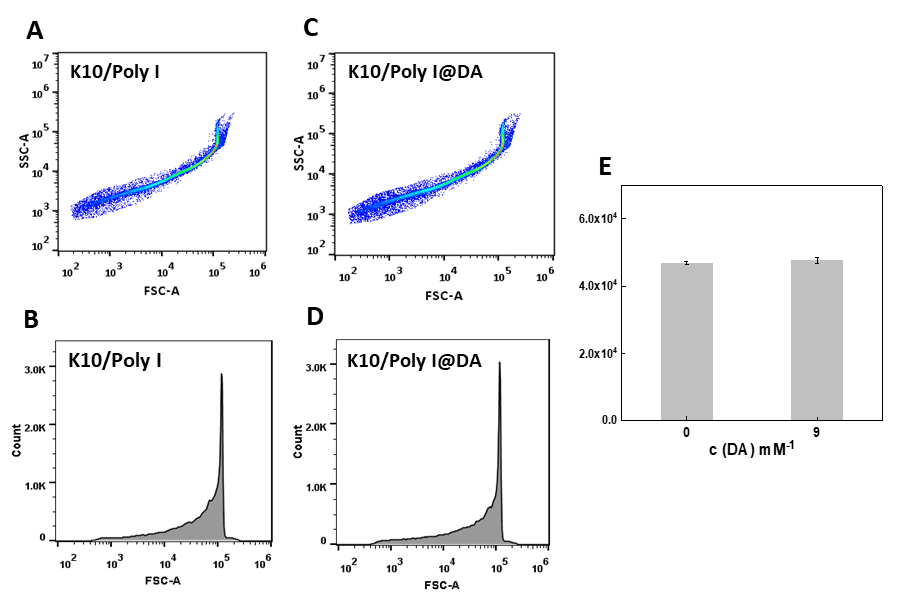


**Figure S3**. (**A-E**) Two-dimensional (2D) dot plots of side-scattered (SSC) versus forward-scattered (FSC) light for non-coated (**A**) and DA-coated (**C**) K10/Poly I biomolecular condensates in aqueous solution and its corresponding histogram plots (**B**, **D**) obtained by FACS. And its corresponding mean condensate microdroplet counts for K10/Poly I biomolecular condensates suspension before and after the addition of decanoic acid (final concentration, 9 mM) (**E**).


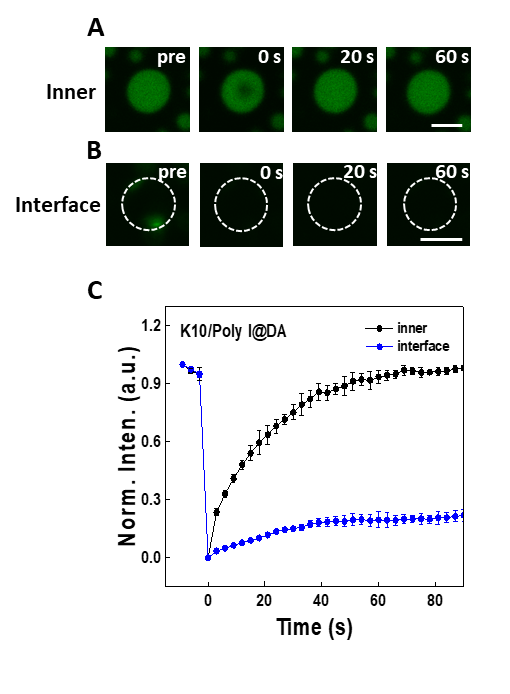


**Figure S4**. (**A-C**) Confocal microscopy images of FRAP measurements showing DA-coated K10/Poly I biomolecular condensates with bleaching area at inner (**A**) or at interface (**B**) of the coacervate microdroplets. Scale bars: 1 μm. (**C**) Normalized intensity of the fluorescence recovery after photobleaching of DA-coated K10/Poly I biomolecular condensates. The blue and black plots correspond to the FRAP measurements recorded at the internal part and at the interface of DA-coated K10/Poly I biomolecular condensates. For coated- K10/Poly I condensates microdroplets, DiO (interface) was excited with a laser at 488 nm. Methylene blue (inner part) was excited with a laser at 640 nm.


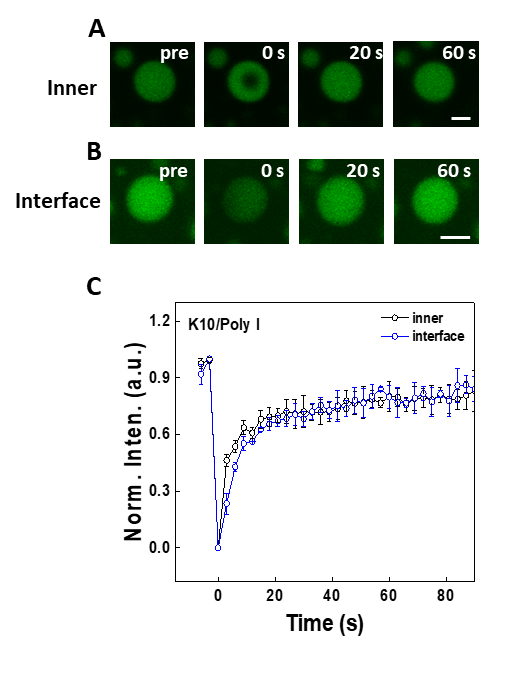


**Figure S5**. (**A-C**) Confocal microscopy images of FRAP measurements showing K10/Poly I biomolecular condensates with bleaching area at inner (**A**) or at interface (**B**) of the coacervate microdroplets. Scale bars: 1 μm. (**C**) Normalized intensity of the fluorescence recovery after photobleaching of K10/Poly I biomolecular condensates. The blue and black plots correspond to the FRAP measurements recorded at the internal part and at the interface of K10/Poly I biomolecular condensates. For K10/Poly I condensates microdroplets, Methylene blue (inner part and interface part) was excited with a laser at 640 nm.


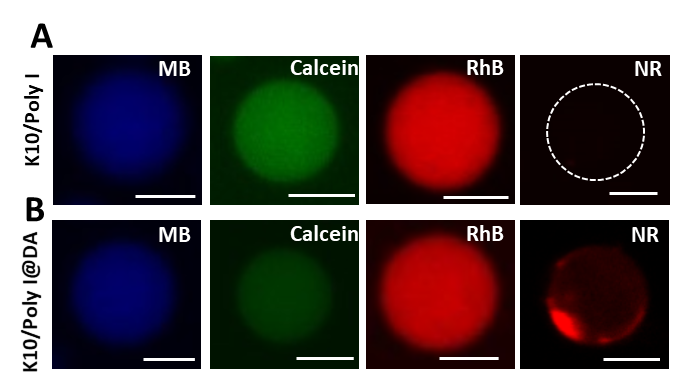


**Figure S6**. (**A, B**) Confocal fluorescence microscopy images recorded after addition of methylene blue (MB), calcein, and rhodamine B (RhB) to K10/Poly I biomolecular condensates (top row) (**A**) and K10/Poly I biomolecular condensates with decanoic acid (final concentration, 9 mm) (bottom row) (**B**). All scale bars: 2 μm.


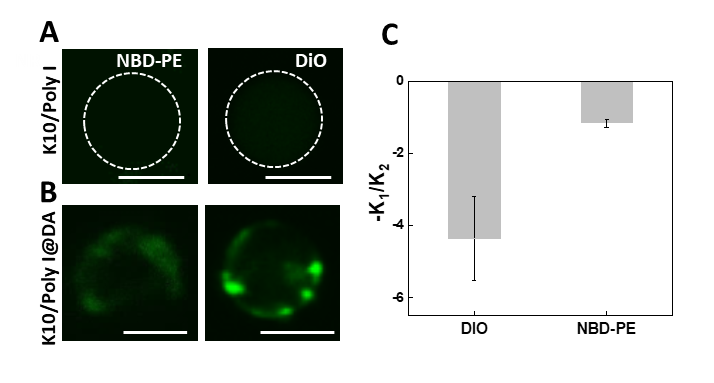


**Figure S7**. (**A-C**) Confocal fluorescence microscopy images recorded after addition of NBD-PE, and DiO to K10/Poly I biomolecular condensates (top row) (**A**) and K10/Poly I biomolecular condensates with decanoic acid (final concentration, 9 mm) (bottom row) (**B**). All scale bars: 2 μm. (**C**) Its corresponding plots of the changes of partitioning coefficients (**C**) before and after the additional of DA. K1 represent partitioning coefficient in DA-coated condensates, and K2 represent partitioning coefficient in non-coated condensates. The difference in the change of partitioning coefficient was obtained by K2/K1 when more dyes were sequestered in non-coated condensates, or by -K1/K2 when more dyes were sequestered in DA-coated condensates.


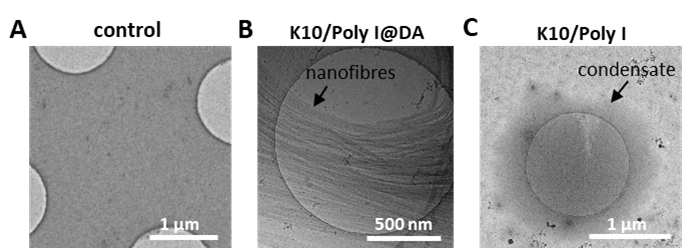


**Figure S8**. (**A-C**) Cryo-TEM images of Fmoc-TyrP (with a final concentration of 2.4 mM) before (**A**) and after incubated with DA-coated (**B**), non-coated (**C**) K10/Poly I condensates for 15 min. Scale bar: 1 μm for (**A**, **C**) and 500 nm for (**B**).


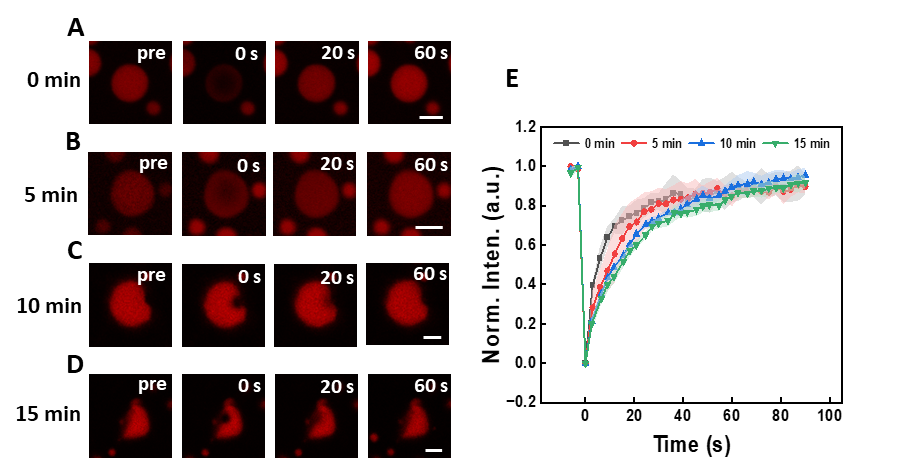


**Figure S9**. (**A-C**) Confocal microscopy images of FRAP measurements showing at 0 (**A**), 5 (**B**), 10 (**C**), and 15 min (**D**) after addition of Fmoc-TyrP to ALP-containing DA-coated K10/Poly I biomolecular condensates. Scale bars: 1 μm. (**E**) Its corresponding normalized intensity of the fluorescence recovery after photobleaching of ALP-containing DA-coated K10/Poly I biomolecular condensates. The grey, red, blue and green plots correspond to the FRAP measurements recorded at 0 (**A**), 5 (**B**), 10 (**C**), and 15 min (**D**) after addition of Fmoc-TyrP to ALP-containing DA-coated K10/Poly I biomolecular condensates. For ALP-containing DA-coated K10/Poly I biomolecular condensates TAMRA-K10 (2 μΜ) was excited with a laser at 561 nm with emission wavelength 560-670 nm.


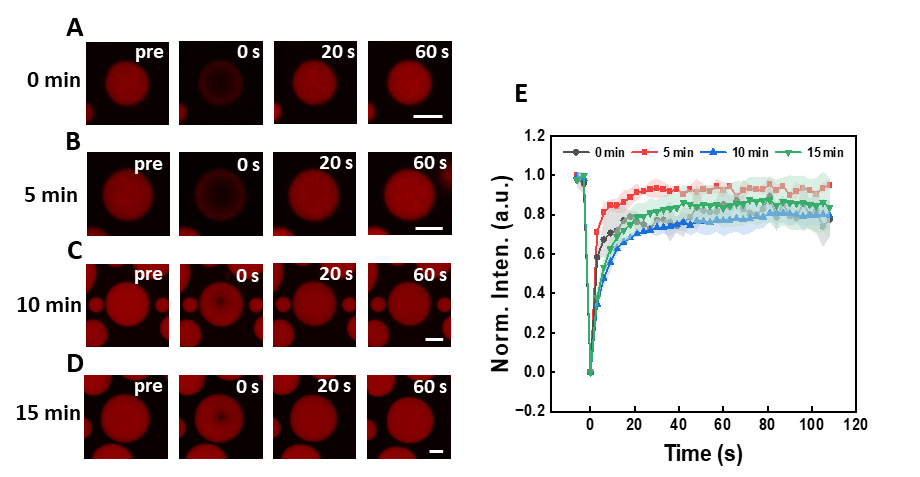


**Figure S10**. (**A-C**) Confocal microscopy images of FRAP measurements showing at 0 (**A**), 5 (**B**), 10 (**C**), and 15 min (**D**) after addition of Fmoc-TyrP to ALP-containing K10/Poly I biomolecular condensates. Scale bars: 1 μm. (**E**) Its corresponding normalized intensity of the fluorescence recovery after photobleaching of ALP-containing K10/Poly I biomolecular condensates. The grey, red, blue and green plots correspond to the FRAP measurements recorded at 0 (**A**), 5 (**B**), 10 (**C**), and 15 min (**D**) after addition of Fmoc-TyrP to ALP-containing K10/Poly I biomolecular condensates. For ALP-containing K10/Poly I biomolecular condensates TAMRA-K10 (2 μΜ) was excited with a laser at 561 nm with emission wavelength 560-670 nm.


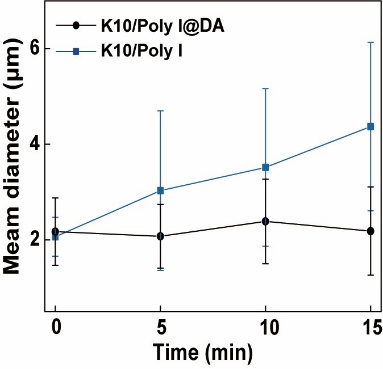


**Figure S11**. Time-dependent quantitative analysis of the mean diameter plots of DA-coated K10/Poly I condensates and non-coated K10/Poly I condensates. Measurements were performed on 20 condensates, and the average value and standard deviation calculated.

*
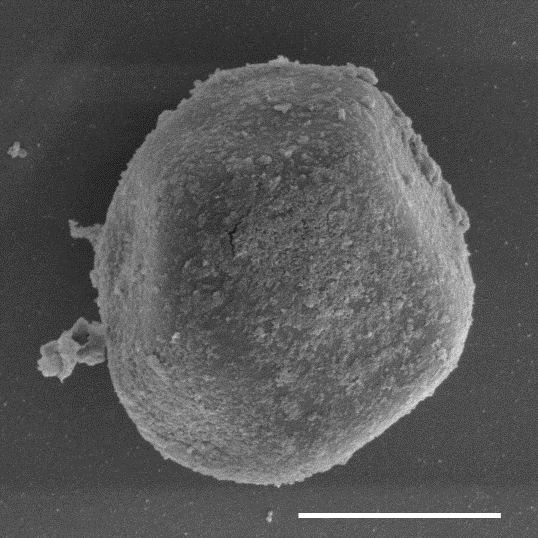
*

**Figure S12**. SEM image of untreated HeLa cell. Scale bar: 5 μm.


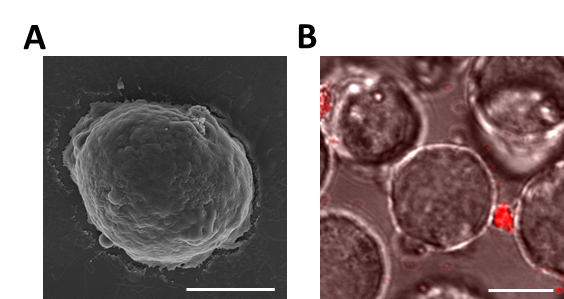


**Figure S13**. (**A**) SEM image of HeLa cell treated by non-coated K10/Poly I biomolecular condensates. Scale bar: 5 μm. (**B**) Confocal fluorescence microscopy image of HeLa cells treated by non-coated K10/Poly I biomolecular condensates (TAMRA-K10 labelled, in red). Scale bar: 10 μm.


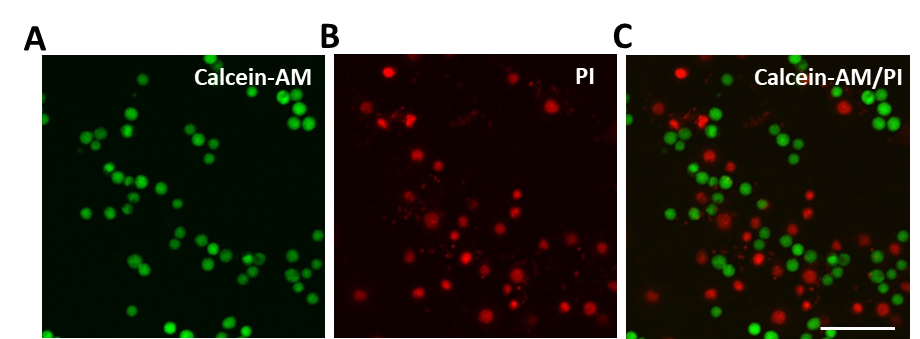


**Figure S14**. (**A-C**) Confocal fluorescence microscopy images recorded at 15 min after addition of Fmoc-TyrP (final concentration, 4.8 mM) to HeLa cells incubated with DA-coated K10/Poly I biomolecular condensates. Scale bar: 100 μm.


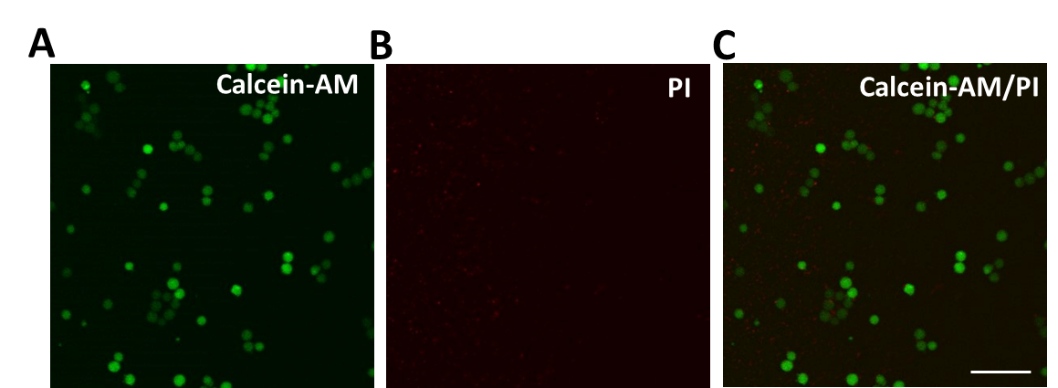


**Figure S15**. (**A-D**) Confocal fluorescence microscopy images of HeLa cells incubated with K10/Poly I biomolecular condensates with decanoic acid (final concentration, 9 mM). DA-coated K10/Poly I biomolecular condensates without Fmoc-TyrP addition show no dead cells. Scale bar: 100 μm.


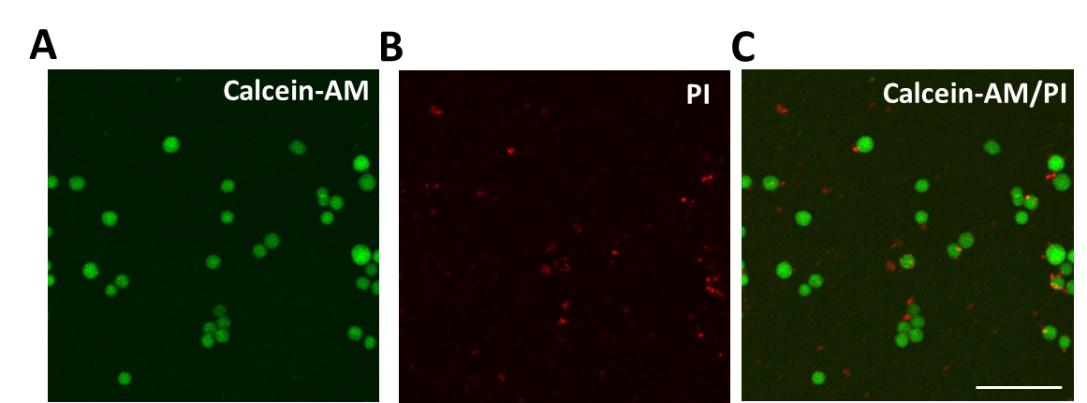


**Figure S16**. (**A-D**) Confocal fluorescence microscopy images recorded at 15 min after addition of Fmoc-TyrP (final concentration, 4.8 mM) to HeLa cells incubated with non-coated K10/Poly I biomolecular condensates. Scale bar: 100 μm.


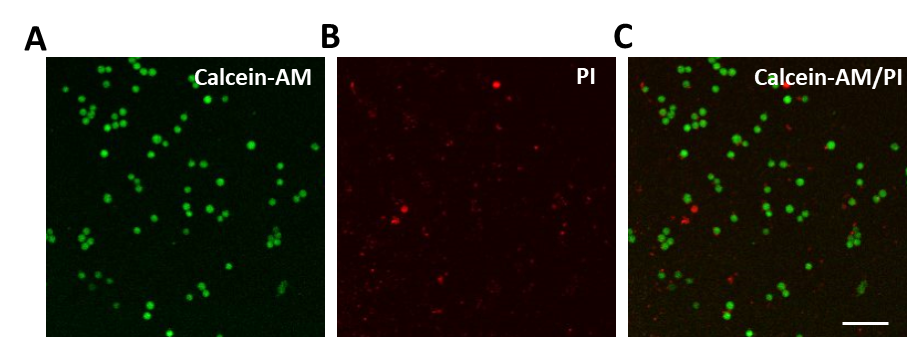


**Figure S17**. (**A-D**) Confocal fluorescence microscopy images recorded at 15 min after addition of Fmoc-TyrP (final concentration, 2.4 mM) to HeLa cells incubated with non-coated K10/Poly I biomolecular condensates. Scale bar: 100 μm.


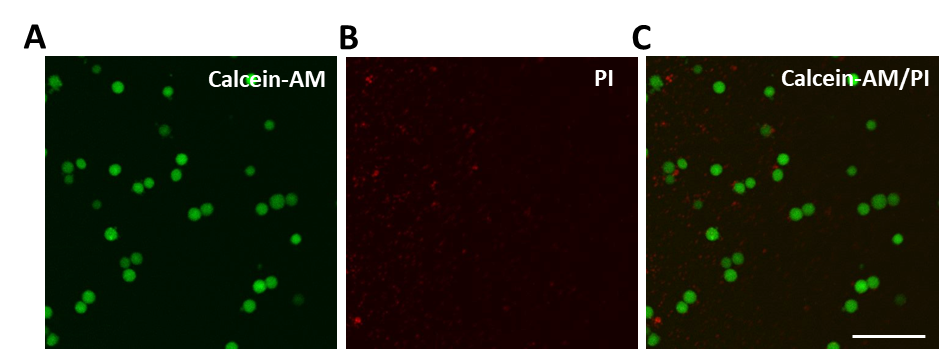


**Figure S18**. (**A-D**) Confocal fluorescence microscopy images recorded at 15 min without Fmoc-TyrP addition to HeLa cells incubated with non-coated K10/Poly I biomolecular condensates. Scale bar: 100 μm.


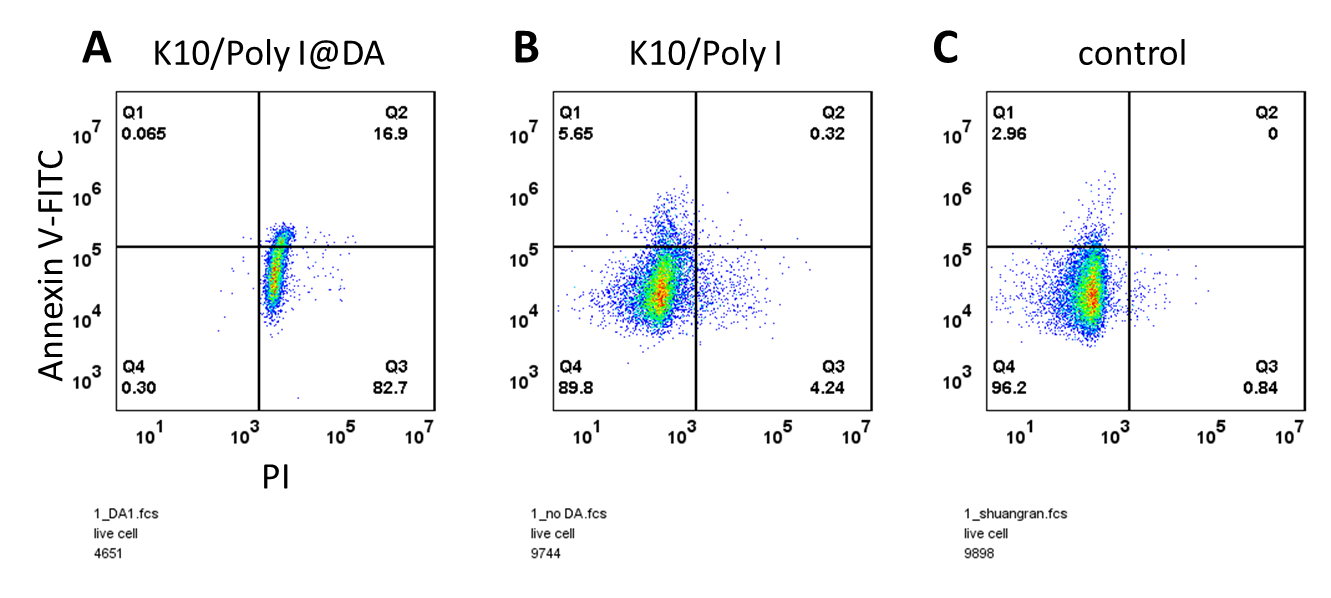


**Figure S19**. (**A**-**C**) The flow cytometry results of the apoptosis of HeLa cells staining with Annexin V-FITC/PI after incubated with DA-coated (**A**), non-coated (**B**) K10/Poly I condensates for 15 min after the addition of Fmoc-TyrP (with a final concentration of 2.4 mM). The enhanced apoptosis promoted by DA-coated K10/Poly I biomolecular condensates confirms the significant effect in killing cancer-cell.
